# Elimination of Ultraviolet Light-Mediated Attraction Behavior in *Culex* Mosquitoes via dsRNA-Mediated Knockdown of Opsins

**DOI:** 10.3390/insects16100997

**Published:** 2025-09-25

**Authors:** Xinyi Liu, Guoqiang Zhao, Hui Liu, Yuxuan Mao, Meng Xu, Jing Wu, Lijiao Li, Zongzhao Zhai, Pa Wu

**Affiliations:** Hunan Provincial Key Laboratory of Animal Intestinal Function and Regulation, College of Life Sciences, Hunan Normal University, Changsha 410081, China; xinyiliu599@sina.com (X.L.); zhaogq@hunnu.edu.cn (G.Z.); 0807lh@sina.com (H.L.); myx2025@sina.com (Y.M.); xumengaa@sina.com (M.X.); jingwu202508@163.com (J.W.); lilijiao202508@163.com (L.L.)

**Keywords:** *Aedes*, *Culex quinquefasciatus*, mosquito, opsin, ultraviolet light

## Abstract

It is widely recognized that mosquitoes are attracted to ultraviolet (UV) light traps in field and semi-field trials. However, the specific characteristics of mosquito behavioral responses to UV light remain poorly defined. Moreover, the molecular mechanisms underlying their phototactic behavior remain unexplored. Here, we characterized mosquito photobehavior under UV light in a laboratory setting using three experimental apparatuses. Our findings indicate that mosquitoes exhibit strong attraction to low-intensity UV light, yet show no preference between high-intensity UV light and darkness. Video recordings and automatic analyses of photobehavior under low-intensity UV light revealed that mosquitoes preferred the window illuminated by UV light over an unilluminated window and were more active when exposed to UV light. Through RNA interference (RNAi)-mediated knockdown of opsins highly expressed in the adult stage of *Culex quinquefasciatus*, we identified *CqOpsin3*, *CqOpsin5*, and *CqOpsin6* as crucial mediators of UV phototaxis. This study provides methods for characterizing mosquito photobehavior under UV light in the laboratory, and represents the first mechanistic investigation into UV light-mediated attractive behavior in mosquitoes.

## Simple Summary

Mosquitoes use ultraviolet light as a navigation cue, but the detailed behavioral characteristics and molecular mechanisms underlying this response remain poorly understood. In this study, we examined how mosquitoes respond to ultraviolet light under controlled laboratory conditions using three distinct experimental assays. We found that mosquitoes were strongly attracted to low-intensity ultraviolet light, but showed no preference between high-intensity ultraviolet light and darkness. Using video tracking and automated computational analysis of their movement, we further demonstrated that mosquitoes were more active under ultraviolet light and preferred areas illuminated with low-intensity ultraviolet light over dark regions. Through intrathoracic injection of dsRNA, we identified three genes essential for mosquito attraction to ultraviolet light. These results reveal, for the first time, the genetic mechanisms governing mosquito attraction to ultraviolet light. Our findings not only provide deeper insight into mosquito light-guided behavior but may also improve the design of light-based control strategies, opening new avenues for reducing mosquito populations and curbing disease transmission.

## 1. Introduction

Mosquitoes pose major public health problems worldwide by transmitting a variety of arboviruses, including dengue virus, Zika virus, yellow fever virus, and Japanese encephalitis virus [1,2]. Light traps, commonly used in field surveillance to monitor mosquito populations, are also frequently employed in households for mosquito control and to reduce arbovirus transmission [3,4]. Despite the widespread use of visible light traps to capture mosquitoes in the field, Liu, et al. [5] revealed that both diurnal and nocturnal mosquitoes are photonegative to visible light, a finding supported by other studies that recommend its removal from light traps [6,7]. Ultraviolet (UV) light, which lies beyond the human visual spectrum (also known as black light), can be perceived by many insects including mosquitoes [8]. Although UV light traps are widely used in field collections, mosquito photobehavior under UV light remains poorly understood.

In *Drosophila*, the behavioral responses to UV and visible light, as well as their interaction, have been extensively studied under controlled conditions [9,10]. In contrast, studies on mosquito responses to light have primarily relied on field-based methods, evaluating trapping efficiency by comparing the catch rates of light traps randomly placed in the field or semi-field conditions [3,11,12,13]. Recent studies have characterized mosquito behavioral responses to the visible spectrum under laboratory conditions. A wind-tunnel assay has been used to investigate the visual responses of *Aedes* mosquitoes to black objects in combination with other sensory cues including odor [14]. Our previous study further characterized the behavioral responses of *Aedes* and *Culex* mosquitoes to white light using an experimental apparatus modified from *Drosophila* photobehavior assay [5]. Despite these advances in studying visible light, mosquito behavioral responses to UV light under controlled laboratory conditions remain relatively poorly understood. Using a custom-designed arena, one study compared the circadian regulation of UV light-evoked attraction and avoidance behaviors in *Aedes* and *Anopheles* mosquitoes, but did not include *Culex* species [15]. The immediate behavioral responses of mosquitoes to UV light, as well as the specific sensory receptors mediating these responses, remain to be elucidated.

Bioinformatic analyses of assembled mosquito genomes, including those of *Aedes aegypti* (*Ae. aegypti*), *Anopheles gambiae*, and *Culex quinquefasciatus* (*Cx. quinquefasciatus*)*,* have predicted a range of opsin genes and their potential sensitive wavelengths [16,17]. In *Cx. quinquefasciatus,* thirteen opsin genes have been identified by bioinformatic analysis, including eight predicted to be long-wavelength-sensitive, one UV-sensitive, and one short-wavelength-sensitive [16]. Molecular evolutionary studies further revealed that seven long-wavelength opsins in *Cx. quinquefasciatus* are homologous to the *Drosophila* Rh6 gene, likely resulting from a series of duplications and diversification [17]. However, among the mosquitoes in the Culicidae family analyzed, only one opsin in each mosquito species is homologous to the UV-sensitive Rh3 and Rh4 genes in *Drosophila* [17]. In addition to bioinformatic analyses, studies have systematically mapped the expression patterns of opsin genes in *Ae. aegypti* [18,19,20,21]. For instance, *Opsin1* is broadly expressed in R1–6 and R8 photoreceptors, while *Opsin2* and *Opsin8* are expressed in a non-overlapping pattern in R7 photoreceptor cells [20,21]. Additionally, *Opsin9* is co-expressed with either *Opsin2* or *Opsin8* in all R7 cells, as well as a subset of R8 cells [18]. Beyond expression profiling, functional studies using gene perturbation approaches have begun to dissect the roles of opsins in the behavioral response to visible light in *Aedes* mosquitoes [5,14]. Despite these advances, significant knowledge gaps remain. The expression patterns and molecular functions of opsin genes in *Culex* mosquitoes remain unexplored. Moreover, the molecular mechanisms underlying UV light perception remain uncharacterized across all mosquito species.

In this study, we characterized the photobehavior of *Cx. quinquefasciatus* under a gradient of UV light intensities, and identified *CqOpsin3*, *CqOpsin5*, and *CqOpsin6* as crucial factors in controlling its phototactic behavior toward UV light.

## 2. Materials and Methods

### 2.1. Mosquitoes

Mosquito colonies of *Aedes albopictus* (*Ae. albopictus*) (eggs purchased from Guangzhou Wolbaki Biotechnology Co., Ltd., Guangzhou, China), *Cx. quinquefasciatus* (eggs kindly provided by Y. Huang, Hunan Provincial Center for Disease Control and Prevention), and the Liverpool strain of *Ae. aegypti* (LVP, obtained from Prof. G.H. Wang, Institute of Zoology, Chinese Academy of Sciences, Beijing, China) were maintained under standard insectary conditions. Larvae were maintained in dechlorinated tap water and fed daily with a sterile liver powder suspension (60 g/L; CM0077, Oxoid, Tokyo, Japan). Pupae were collected using transfer pipettes and placed in bowls inside mesh cages for adult emergence. Adults were housed in 40 cm × 40 cm × 40 cm mesh cages with unlimited access to water and sugar sources (raisins). All developmental stages were maintained in incubators (HWS-1000, Ningbojiangnan, Ningbo, China) under standard insectary conditions (27 °C, 75% relative humidity, 12 h:12 h light/dark cycle). All behavioral assays were conducted with 4–10 days post-emergence adult female mosquitoes, as females are the host-seeking sex responsible for blood feeding and virus transmission, making them the biologically relevant target for investigating UV light attraction.

Based on the established ecological and morphological traits of *Cx. quinquefasciatus*, we conducted field collections targeting typical larval habitats of this species. Specifically, we surveyed sewage ditches, contaminated puddles, septic tanks, fertilizing buckets, and other organically polluted aquatic environments located in and around campus areas, as well as in nearby villages and farmland. Larvae and pupae were collected from these sites using a sieve, transferred to plastic containers, and transported to the laboratory. After emergence, adults were identified as *Cx. quinquefasciatus* based on key morphological characteristics and kept in mesh cages with unlimited access to water and sugar (raisins). All field-collected mosquitoes were maintained in a room exposed to natural sunlight through a window. The ambient temperature and humidity in the room were similar to outdoor conditions.

### 2.2. Mosquito Photobehavior

#### 2.2.1. Y-Maze Photobehavior Assay

**Figure 1:**
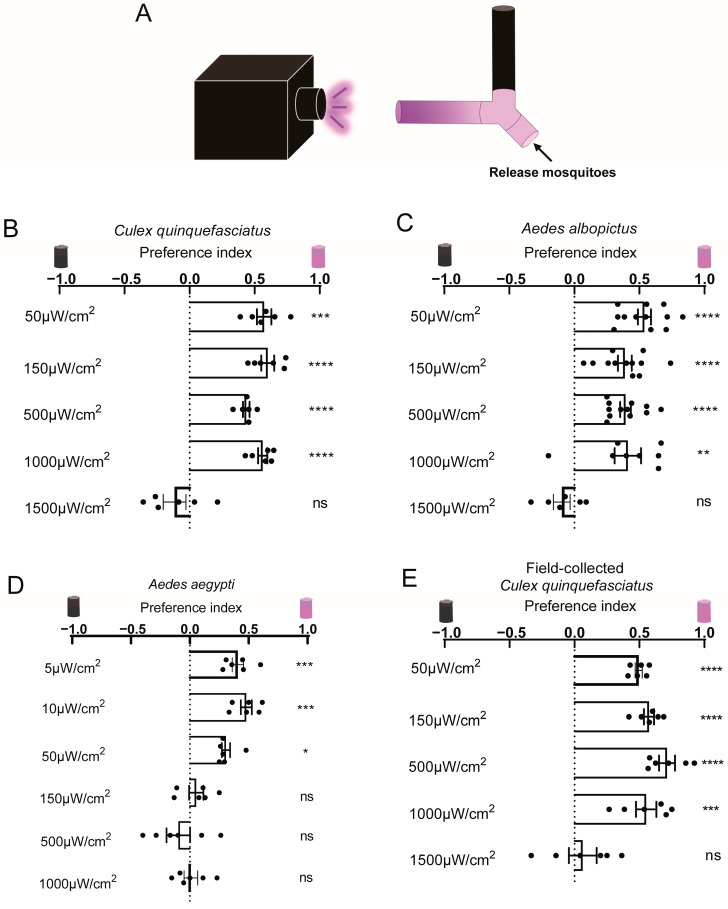
**Phototaxis of female mosquitoes under UV light with a gradient of intensity.** (**A**) Schematic diagram of the Y-maze assay. The arrowhead indicates the mosquito release point. (**B**–**E**) Preference for UV-lit versus shaded area. Behavioral response of laboratory-reared *Cx. quinquefasciatus* (**B**), laboratory-reared *Ae. albopictus* (**C**), laboratory-reared *Ae. aegypti* (**D**) and field-collected *Cx. quinquefasciatus* (**E**) to a gradient of UV light intensities. (**B**–**E**) Values represent mean ± SEM of ≥3 biological replicates (two technical replicates each). The total number of female mosquitoes tested in each group is presented in Table S1. Statistical significance of phototaxis relative to chance was computed using a one-sample *t*-test or Wilcoxon signed-rank test. Significance: * *p* < 0.05, ** *p* < 0.01, *** *p* < 0.001, **** *p* < 0.0001, ns: not significant.

A Y-maze assay, which we previously designed to study mosquito behavioral responses to visible light [5], was adopted to test mosquito photobehavior under UV light. A computer-controlled Tunable Light Source (CME-TLSX300F, Microenery, Beijing, China) provided UV illumination to one of the two long arms of the Y-maze (Figure 1A) with light intensity measured by a UV Radiation Meter (UV340B, Sanpometer, Shenzhen, China). The total number of female mosquitoes tested in each group is presented in Table S1. For each biological replicate, we performed two technical replicates with the light source alternated between the two long arms to control for any arm-specific bias. Mosquitoes that remained in the release arm without making a choice were excluded from analysis. The preference index (PI) was calculated as follows: PI = (Number of mosquitoes in the UV illuminated arm − Number of mosquitoes in darkness)/(Number of mosquitoes in the UV illuminated arm + Number of mosquitoes in darkness). PI > 0 indicates that more mosquitoes preferred the UV illuminated area, while the opposite is true for PI < 0.

#### 2.2.2. Tube Photobehavior Assay

**Figure 2:**
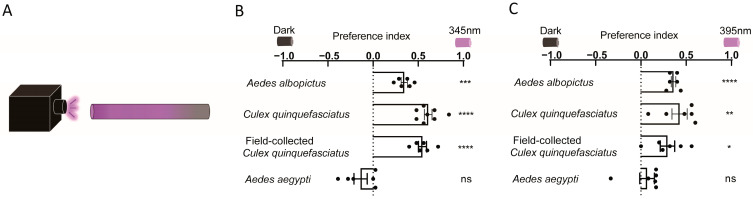
**Phototaxis of female mosquitoes under UV light of different wavelengths.** (**A**) Schematic diagram of the tube assay. (**B**,**C**) Assessment of mosquito behavioral response to UV light using a tube assay. Mosquito photobehavior under 345 nm (**B**) and 395 nm (**C**) UV light at an intensity of 50 μW/cm^2^. (**B**,**C**) Values represent mean ± SEM of ≥3 biological replicates (two technical replicates each). The total number of female mosquitoes tested in each group is presented in Table S1. Statistical significance of phototaxis relative to chance was computed using a one-sample *t*-test or Wilcoxon signed-rank test. Significance: * *p* < 0.05, ** *p* < 0.01, *** *p* < 0.001, **** *p* < 0.0001, ns: not significant.

This assay was modified from a previously established tube assay [5] designed to investigate mosquito photobehavior under visible light, using the same experimental apparatus. While overhead white LED light provided illumination through the glass tube walls in the previous assay [5], UV light was directionally introduced from one open end of the glass tube without passing through the glass wall in this assay (Figure 2A). Prior to the experiment, mosquitoes were released into the glass tube and allowed to acclimate for 10 min (min), during which they distributed evenly within the tube. After the acclimation period, mosquitoes were exposed to UV light and allowed 5 min to make their choice. Light intensity was measured with a UV Radiation Meter (UV340B, Sanpometer, Shenzhen, China). PI = (N_near − N_far)/(N_near + N_far), where N_near represents the number of mosquitoes in the half of the tube closest to the UV light source, and N_far represents the number of mosquitoes in the half of the tube farthest from the UV light source. PI > 0 indicates that more mosquitoes preferred the UV illuminated area, while the inverse is true for PI < 0.

#### 2.2.3. Automatic Analysis of Mosquito Photobehavior

**Figure 3:**
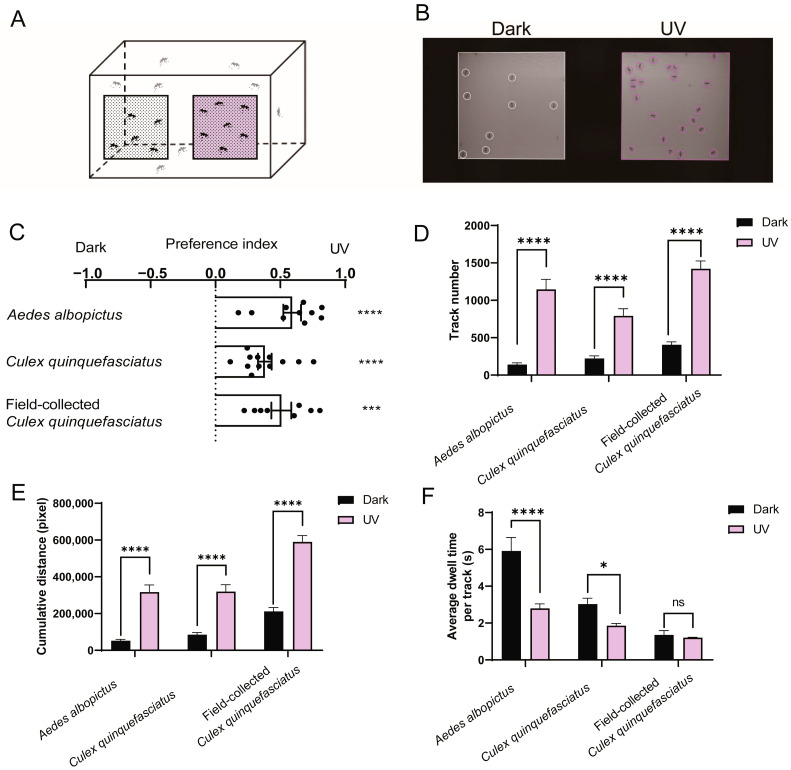
**Video recording and automatic analysis of mosquito photobehavior under UV light.** (**A**) Schematic diagram of video recording setup. Mosquitoes were exposed to 345 nm UV light from one of the two meshed windows, and their behavior was video recorded for 2 min. (**B**) Representative video frame taken from an experiment in which females were exposed to 345 nm UV light at 50 μW/cm^2^. (**C**–**F**) Photo preference between darkness and 345 nm UV light at 50 μW/cm^2^. Shown are the preference index (**C**), the total number of mosquito tracks (**D**), the cumulative distance moved by mosquitoes (**E**) and the average dwell time per mosquito track (**F**) on UV-lit versus unlit mesh window. (**C**–**F**) Values represent mean ± SEM of ≥3 biological replicates (two technical replicates each). The total number of female mosquitoes tested in each group is presented in Table S1. (**C**) Data were analyzed using one sample *t*-test. (**D**–**F**) Group comparisons were performed using unpaired *t*-test. Significance: * *p* < 0.05, *** *p* < 0.001, **** *p* < 0.0001, ns: not significant.

This assay was designed to allow automated object-tracking analysis. The test cage was composed of acrylic panels measuring 30 cm × 10 cm × 15 cm (length × width × height) and featured two meshed windows (9 cm × 9 cm) on one panel (Figure 3A). UV light at 345 nm, emitted from a computer-controlled Tunable Light Source (CME-TLSX300F, Microenery, Beijing, China) provided illumination for one of the two meshed windows. To control for positional bias, the meshed window illuminated by UV light was alternated after each trial. The white opaque test cage contained one transparent acrylic panel on the top to allow infrared light of 850 nm (SMD2835-300, Ledlightsworld, Shenzhen, China) to provide illumination for video recording in darkness. A camera (a2A1920-160 μm BAS, Basler, Ahrensburg, Germany) equipped with an infrared filter (central wavelength of 850 nm, BP850-35.5, Basler, Germany) was positioned opposite the meshed window. Recordings were made at 1920 × 800 resolution and 25 fps with manual focus and exposure settings using Pylon Viewer 8.0.0 software. Female mosquitoes were acclimated in the test cage for at least 24 h before testing with continuous access to water and sugar (raisins). For the photobehavior assay, each trial involved the release of eighty untreated mosquitoes. Mosquitoes were exposed to UV light of the indicated intensity and wavelength from a computer-controlled Tunable Light Source (CME-TLSX300F, Microenery, Beijing, China) at one of the two meshed windows for 2 min. Following a 30 min rest period in darkness, a technical replication was performed with the UV light illumination switched to the other window. To ensure consistency and minimize circadian variability, all behavioral assays were conducted between Zeitgeber Time (ZT) 6 and ZT12 for *Cx. quinquefasciatus*, and between ZT1 and ZT10 for *Ae. albopictus* and *Ae. aegypti*.

We used an automated object-tracking and preference index calculation script based on MATLAB (R2022b, MathWorks, Natick, MA, USA), modified from Chandel, et al. [22], to analyze mosquitoes in recorded video. The workflow of the object-tracking script includes the following steps: (1) reading the recorded video and manually selecting two regions of interest (corresponding to the two meshed windows); (2) randomly extracting 100 frames from the video to calculate the modal pixel value, which is used to generate a background model; (3) identifying mosquitoes using the background model and a white-and-black model. The background model identifies moving objects by analyzing the absolute differences in pixel values between each frame and the background. The white-and-black model detects stationary mosquitoes which appear as black blobs against the white background; (4) assigning IDs to each tracked mosquito for motion analysis; (5) calculating the PI and other behavioral metrics. The overall PI for a given time period was calculated as the average of the PI values from each frame. For each frame, PI was defined as: PI = (Number of mosquitoes on UV-illuminated window − Number of mosquitoes on the window in darkness)/(Number of mosquitoes on UV-illuminated window + Number of mosquitoes on the window in darkness). PI > 0 indicates that more mosquitoes preferred the UV illuminated area, while the inverse is true for PI < 0.

Track number was calculated as the sum of IDs in the respective window within the 2 min recorded time. The cumulative distance mosquitoes moved on a given mesh window was calculated as the sum of the distances traveled by each track on that window. The distance of each track was calculated as the sum of the Euclidean distances between two adjacent coordinates of the track. The average dwell time per track was calculated by dividing the total time spent by all mosquitoes in that window by the number of mosquito tracks. The total time spent by each mosquito in a respective window was calculated by dividing the number of video frames the mosquito spent in that window by the video frame rate.

### 2.3. RNA Extraction and Quantification of Gene Expression

The RNA expression profile of *Cx. quinquefasciatus* and *Ae. aegypti* were analyzed at selected developmental stages, including fourth-instar larvae, pupae and adults aged 5 to 7 days post-emergence. Tissues from these stages were homogenized for RNA extraction. Female adult mosquitoes on the third day post double-strand RNA (dsRNA) injection were also homogenized for RNA extraction. RNA extraction and complementary DNA (cDNA) synthesis were performed using RNAiso Plus (9190, Takara, Beijing, China) and the PrimeScript RT reagent kit (RR037A, Takara, Beijing, China), respectively. To analyze opsin gene expression in *Ae. albopictus* and *Cx. quinquefasciatus*, RNA samples for quantitative real-time polymerase chain reaction (RT-qPCR) were collected within the same circadian time window as that used for behavioral assays.

Gene expression was analyzed by RT-qPCR with the ChamQ SYBR Color qPCR Master Mix (Q421, Vazyme, Nanjing, China) on a Quantagene q225 thermal cycler system (q225, Kubo Technology, Beijing, China). The primers for RT-qPCR are listed in Supplementary Table S2. Gene expression levels were normalized to the reference genes Rpl8 (*LOC6031076*) in *Cx. quinquefasciatus* and actin (*AAEL011197*) in *Ae. aegypti*.

### 2.4. Gene Silencing in Mosquitoes

Gene silencing was performed using synthetized and purified dsRNA prepared with the MEGAscript T7 transcription kit (AM1334, Invitrogen, Carlsbad, CA, USA) according to the manufacturer’s protocol. Female adult mosquitoes, aged 0–5 days post-eclosion and not blood-fed, were anesthetized at 4 °C and placed on a CO_2_ gas plate. Using the Glass Micromotor Syringe Pump (R-480, RWD, Shenzhen, China), 300 nL of solution containing 1 μg of dsRNA was injected into the thorax of each mosquito. Behavioral assays were conducted 3 days post-injection, and silencing efficiency was validated by RT-qPCR. Mosquitoes injected with double-strand green fluorescent protein (dsGFP) were used as the control group. The primers used in this assay are listed in Supplementary Table S3.

### 2.5. Statistical Analysis

Statistical analyses and graphing were performed using GraphPad Prism 8 (Prism, La Jolla, CA, USA). A schematic summary of assay type, mosquito species, light wavelength, light intensity, biological replicates, total number of mosquitoes tested, and test duration is provided in Supplementary Table S1. All datasets were assessed for normality. To determine whether PI significantly differed from chance, we applied a one-sample *t*-test (for normal distribution data) or a Wilcoxon signed-rank test (for non-normal distributions). Comparisons between two groups were conducted using an unpaired *t*-test (normal distribution) or a Mann–Whitney test (non-normal distribution). Multiple group comparisons were performed using one-way ANOVA with Tukey’s post hoc test (normal distribution) or a Kruskal–Wallis test with Dunn’s post hoc test (non-normal distribution). The *p* value was used to evaluate the null hypothesis that any observed effect was due to chance or that there was no difference between the means of the two groups. Statistical significance was set at the level of specifically * *p* < 0.05, ** *p* < 0.01, *** *p* < 0.001, **** *p* < 0.0001, ns: not significant. Details of the test methods are summarized in Tables S4 and S5. In figures, datasets labeled with different lowercase letters indicate statistically significant differences between groups.

### 2.6. Ethical Note

The study has been approved by the Hunan Normal University Ethics Committee (Protocol 188/2022).

## 3. Results

### 3.1. Mosquitoes Preferred Low Intensity UV over Darkness

Previous studies have assessed mosquito responses to UV light using light traps in field or semi-field trials by evaluating attraction efficiency based on the number of mosquitoes caught per trap [3,11,12,13]. However, to investigate the mechanisms underlying mosquito phototaxis in response to UV light, a controlled laboratory assay needs to be established. We employed a Y-maze assay (Figure 1A), adapted from a well-established *Drosophila* light/dark assay used to study photobehavior [23], to characterize mosquito photobehavior under UV light. The preference index was calculated following an approach similar to that used in *Drosophila* phototaxis studies [9]: PI = (N_UV_ − N_D_)/(N_UV_ + N_D_), where N_UV_ and N_D_ represent the number of mosquitoes on the UV-illuminated side and the dark side, respectively. We conducted a Y-maze assay in which mosquitoes could choose between darkness and a gradient of UV light intensities. Laboratory-reared *Cx. quinquefasciatus* and *Ae. albopictus* consistently preferred UV light at intensities ranging from 50 μW/cm^2^ to 1000 μW/cm^2^ over darkness, but did not show a significant preference between darkness and UV light at 1500 μW/cm^2^ (Figure 1B,C). The *Ae. aegypti* Liverpool strain (LVP) was more sensitive to UV light, preferring intensities between 5 μW/cm^2^ and 50 μW/cm^2^ over darkness, but exhibited no significant preference between darkness and UV light at 150 μW/cm^2^, 500 μW/cm^2^, and 1000 μW/cm^2^ (Figure 1D). While UV light is widely used for mosquito trapping in the field, we investigated whether field-collected mosquitoes displayed similar photobehavior. We collected larvae and pupae of *Cx. quinquefasciatus* from the field and tested the emerged adults using the Y-maze assay. Consistently, field-collected *Cx. quinquefasciatus* preferred low-intensity UV light below 1000 μW/cm^2^, but no significant phototaxis was observed at 1500 μW/cm^2^ (Figure 1E). These results indicate that mosquito phototaxis is affected by illumination intensity. Accordingly, subsequent experiments with *Cx. quinquefasciatus* and *Ae. albopictus* were conducted using UV light intensities below 1000 μW/cm^2^, while *Ae. aegypti* was tested at 50 μW/cm^2^.

To further validate the observation that mosquitoes exhibit positive phototaxis toward low-intensity UV light, we conducted a tube assay using a 60 cm glass tube illuminated by UV light at one end (Figure 2A). UV light at 50 μW/cm^2^ attracted *Cx. quinquefasciatus* and *Ae. albopictus* to the near-UV end of the tube but did not affect the distribution of *Ae. aegypti* (Figure 2B). Subsequently, we tested UV light at 150 μW/cm^2^ measured at the near-UV end of the tube. Consistently, *Cx. quinquefasciatus* and *Ae. albopictus* that were initially randomly distributed in the tube moved toward the UV light within 5 min after light exposure (Figure S1A). While the abovementioned experiments evaluated mosquito phototaxis with 345 nm UV light, we further investigated mosquito photobehavior under 395 nm UV light. When tested with a Y-maze assay, all three mosquito species preferred the UV light-illuminated environment at 395 nm over an unilluminated one (Figure S1B). In the tube assay, 395 nm UV light of 50 μW/cm^2^ attracted *Cx. quinquefasciatus* and *Ae. albopictus* to the near-UV end of the tube, whereas it failed to modulate the distribution of *Ae. aegypti* (Figure 2C). Collectively, these results demonstrated that *Cx. quinquefasciatus* and *Ae. albopictus* were significantly attracted to low-intensity UV light across different spectra and experimental setups. By contrast, the behavioral response of *Ae. aegypti* to UV light was context-dependent.

### 3.2. UV Light Affects Mosquito Preference and Activity

To further illustrate mosquito photobehavior under UV light, we developed an additional behavioral assay to enable video recording and automated analysis of mosquito behavior. Custom-designed opaque acrylic cages with two mesh windows on one panel (Figure 3A) were used for subsequent study in a dark room. Eighty acclimatized female mosquitoes, housed in the assay cage for over 24 h, were exposed to UV light (50 μW/cm^2^) emanating from one of the two windows for 2 min, while their behavior was recorded (Figure 3B and Supplementary Video S1). We used a custom video-tracking program modified from a previous study [22] to automatically analyze mosquito behavior. Compared with traditional manual measurement at set intervals, the automated program enabled continuous recording and analysis of mosquito activity throughout the 2 min experimental window (Supplementary Video S2). We calculated the average PI for the 2 min video by summing the PI of each frame and dividing by the total number of frames, with PI of each frame calculated as PI = (N_UV_ − N_D_)/ (N_UV_ + N_D_), where N_UV_ and N_D_ represent the number of mosquitoes on the UV-illuminated side and the dark side, respectively. Consistent with the Y-maze assay, the results showed that mosquitoes significantly preferred the UV-illuminated area over the unilluminated area (Figure 3C). To further characterize mosquito photobehavior under UV light, we calculated three additional metrics: the number of mosquito tracks at each window, the cumulative distance mosquitoes moved on the respective mesh window across all tracks, and the average dwell time per mosquito track on the respective mesh window. The number of mosquito tracks as well as the total distance of tracks in the UV-illuminated window was significantly larger compared to those in the unilluminated window (Figure 3D,E). By contrast, laboratory-reared *Ae. albopictus* and *Cx. quinquefasciatus* mosquitoes spent significantly longer time per track in darkness than at the UV-illuminated window (Figure 3F). These results suggest that mosquitoes were more active under UV illumination, while mosquitoes in darkness were predominantly stationary. UV light of 150 μW/cm^2^ drew similar conclusions (Figure S2). Collectively, our results show that mosquitoes preferred UV light and exhibited enhanced activity.

### 3.3. CqOpsin3, CqOpsin5, and CqOpsin6 Mediate Phototactic Behavior to UV Light in Cx. quinquefasciatus

**Figure 4:**
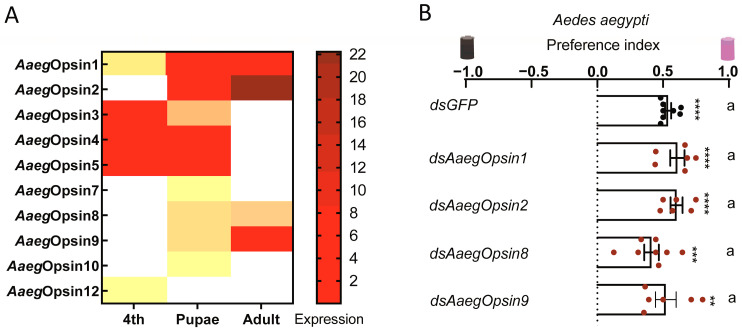
**Photobehavior of opsin-silenced *Ae. aegypti.*** (**A**) Expression of *Ae. aegypti* opsin genes in fourth-instar larvae (4th), pupae, and adults, normalized to *Ae. aegypti* Actin (*AAEL011197*). (**B**) The photobehavior of opsin-silenced *Ae. aegypti* assessed using a Y-maze assay under 345 nm UV light at an intensity of 10 μW/cm^2^. Values represent mean ± SEM of ≥3 biological replicates (two technical replicates each). The total number of female mosquitoes tested in each group is presented in Table S1. Statistical significance of phototaxis relative to chance was computed using a one-sample *t*-test or Wilcoxon signed-rank test. Multiple group comparisons were analyzed using the non-parametric Kruskal–Wallis test, followed by Dunn’s test. Significance: ** *p* < 0.01, *** *p* < 0.001, **** *p* < 0.0001. Different lowercase letters indicate statistically significant differences between groups.

Opsins are a class of transmembrane proteins that initiate photon-induced signaling pathways in both invertebrates and vertebrates [16]. Our previous study identified *opsin1* as a critical regulator of photonegative behavior toward visible light [5]. Here, we investigated which opsin regulates the positive phototaxis of *Aedes* mosquitoes toward UV light. RT-qPCR analysis identified four opsin genes highly expressed in the adult stage of *Ae. aegypti* (Figure 4A). We knocked down these four opsins at the transcript level through intrathoracic injection of dsRNA, resulting in a reduction in their relative abundance of over 74% (Figure S3). However, silencing these genes had no significant effect on the photopositive behavior of *Ae. aegypti* toward low-intensity UV light (Figure 4B).

**Figure 5:**
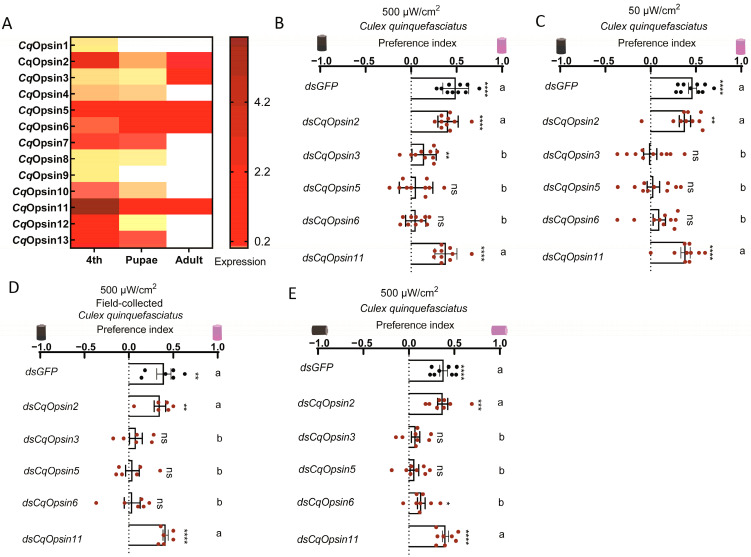
**Photobehavior of opsin-silenced *Cx. quinquefasciatus.*** (**A**) Expression of *Cx. quinquefasciatus* opsin genes in fourth-instar larvae (4th), pupae, and adults, normalized to *Cx. quinquefasciatus* Rpl8 (*LOC6031076*). (**B**–**D**) Photobehavior of opsin-silenced *Cx. quinquefasciatus* assessed using the Y-maze assay. Effects of opsin knockdown on photobehavior of laboratory-reared *Cx. quinquefasciatus* under 345 nm UV light of 500 μW/cm^2^ (**B**) and 50 μW/cm^2^ (**D**). Effects of opsin gene knockdown on the photobehavior of field-collected *Cx. quinquefasciatus* under 345 nm UV light of 500 μW/cm^2^. (**E**) Photobehavior of opsin-silenced *Cx. quinquefasciatus* evaluated using a tube assay under 345 nm UV light at an intensity of 500 μW/cm^2^. (**B**–**E**) Values represent mean ± SEM of ≥3 biological replicates (two technical replicates each). The total number of female mosquitoes tested in each group is presented in Table S1. Statistical significance of phototaxis relative to chance was computed using a one-sample *t*-test or Wilcoxon signed-rank test. Different lowercase letters indicate statistically significant differences between groups. (**B**–**E**) Multiple group comparisons were analyzed using a one-way ANOVA, followed by Tukey’s multiple comparisons test. (**C**) Multiple group comparisons were analyzed using the non-parametric Kruskal–Wallis test, followed by Dunn’s test. Significance: * *p* < 0.05, ** *p* < 0.01, *** *p* < 0.001, **** *p* < 0.0001, ns: not significant.

We next investigated which opsins regulate the positive phototaxis of *Culex* mosquitoes toward UV light. Bioinformatic analysis predicted thirteen opsin genes in *Cx. quinquefasciatus* [16]. To elucidate the mechanisms underlying photopositive responses to low-intensity UV light, we first profiled the expression patterns of these thirteen opsin genes in *Cx. quinquefasciatus* using RT-qPCR (Figure 5A). The results revealed that *CqOpsin2*, *CqOpsin3*, *CqOpsin5*, *CqOpsin6* and *CqOpsin11* were highly expressed in adults (Figure 5A). To assess their functional roles in low-intensity UV light responses, we conducted RNAi-based screening by intrathoracic injection of double-stranded RNA targeting each of the five highly expressed opsins. This approach efficiently knocked down all five opsins at the transcript level, reducing their relative abundance by more than 59% (Figure S4). Knockdown of *CqOpsin5* and *CqOpsin6* completely abolished the photopositive behavior of *Cx. quinquefasciatus* toward UV light at 500 μW/cm^2^, whereas silencing *GFP* (negative control), *CqOpsin2* and *CqOpsin11* had no discernable effect (Figure 5B). Additionally, silencing *CqOpsin3* significantly reduced photopositive behavior under 500 μW/cm^2^ UV light (Figure 5B). Furthermore, knockdown of *CqOpsin3*, *CqOpsin5* and *CqOpsin6* completely abolished the photopositive behavior under 50 μW/cm^2^ UV light (Figure 5C). To validate the roles of these opsins in UV light-mediated attraction in field-derived *Cx. quinquefasciatus*, we silenced the five adult-expressed opsins in adults that had emerged from field-collected larvae and pupae. Consistently, silencing *CqOpsin3*, *CqOpsin5* and *CqOpsin6* significantly abolished the photopositive behavior toward UV light in field-collected mosquitoes (Figure 5D). Moreover, we further confirmed these findings using a tube assay (Figure 5E). Collectively, these results demonstrate that *CqOpsin3*, *CqOpsin5* and *CqOpsin6* play vital roles in UV light-mediated attraction behavior in *Cx. quinquefasciatus*.

## 4. Discussion

It is widely acknowledged that mosquitoes are attracted to UV light; however, the molecular mechanisms underlying this phenomenon remain unexplored. To address this gap, we characterized mosquito photobehavior under UV light using a Y-maze assay and further analyzed their behavior in a test cage with an automatic tracking program. Through dsRNA-mediated knockdown, we identified *CqOpsin3*, *CqOpsin5* and *CqOpsin6* as essential regulators of UV light-induced phototaxis.

Our results showed that UV light at low-intensities is an attractive cue for mosquitoes, whereas exposure to high-intensities of UV light (above 1500 μW/cm^2^) failed to elicit attraction. A comparable phenomenon has been reported in *Drosophila* [24]. It is well-established that adult flies are attracted to UV light within minutes of its onset [25]. However, a recent study indicated that UV light intensity influences the decision between positive phototaxis and avoidance behavior [24]. This avoidance response is thought to reflect an evolutionary adaptation to avoid the detrimental effects of short-wavelength light [24,26].

Another notable finding of our study is that *Ae. aegypti* exhibited heightened sensitivity to UV light, being attracted only at intensities below 50 μW/cm^2^, unlike other tested mosquito species. Although *Ae. aegypti* and *Ae. albopictus* are closely related evolutionarily, with their corresponding opsins sharing high sequence similarity [5], their phototactic responses to UV light between 150 μW/cm^2^ and 1500 μW/cm^2^ diverged significantly. Whether this differential arises from differential opsin expression, variation in neural processing, or contributions from non-visual sensory pathways remains to be elucidated. Indeed, our study is limited in that we did not assess the photobehavior of field-collected *Ae. aegypti,* and all experiments were conducted under controlled laboratory conditions. Further validation of this species-specific behavioral response in the field could provide important theoretical insights for optimizing UV light traps to more precisely target particular mosquito populations.

Phylogenetic analyses predicted *AaegOpsin8* to be UV-sensitive [16]; however, individual knockdown of this gene, as well as each of the other highly expressed adult-stage opsins, did not affect UV attraction in *Ae. aegypti* in our assays. One possible explanation is functional redundancy, whereby another yet unidentified UV-sensitive opsin may compensate for the loss of *AaegOpsin8*. To test this hypothesis, generating double or triple opsin knockout mutants using genetic engineering approaches would be a useful direction for future research.

Bioinformatic analysis predicted only one UV-sensitive opsin in *Cx. quinquefasciatus* [16], whereas our study identified three opsins that are essential for photopositive behavior toward UV light. Although *Opsin5* and *Opsin6* were predicted to be long-wavelength-sensitive opsins [16], our functional data demonstrate their requirement for UV phototaxis; nevertheless, their exact mechanism of action remains to be elucidated. Notably, the *Drosophila* opsin Rh1 exhibits broad-spectrum sensitivity to blue and green light, alongside a secondary peak in the UV range resulting from a bound carotenoid-derived pigment [27,28]. Therefore, characterizing the spectral properties of *Culex* opsins using techniques such as microspectrophotometry (MSP) and electroretinography (ERG) with ectopic expression of each opsin in mosquito photoreceptor cells could provide valuable information on mosquito photobehavior under UV light in addition to our behavioral tests.

Besides opsins, cryptochromes (CRY), which are key regulators of circadian rhythm in response to blue or UV-A light, have also been shown to modulate UV light-evoked avoidance/attraction behavior in *Drosophila* [26,29]. In fruit flies, attraction under low-intensity UV light is primarily mediated by opsins in photoreceptor cells, whereas avoidance under high-intensity UV light is regulated through CRY and Rh7-dependent pathways [24]. Whether similar mechanisms involving cryptochromes and other non-opsin phototransduction molecules influence photobehavior in mosquitoes remains an open question that warrants further investigation.

In conclusion, the behavioral response of mosquitoes to UV light follows a dosage-dependent pattern, highlighting the need to optimize UV intensity for field collections. Our study represents the first mechanistic research into UV light-mediated attractive behavior, revealing the vital role of *CqOpsin3*, *CqOpsin5*, and *CqOpsin6* in UV light phototaxis of *Cx. quinquefasciatus*. These findings not only advance our understanding of mosquito photosensory biology but also provide a functional foundation for developing more efficient and species-specific light-based control strategies.

## Data Availability

The data presented in this study are openly available in the Zenodo data repository at 10.5281/zenodo.14942954, with the URL: https://zenodo.org/records/14942954?preview=1&token=eyJhbGciOiJIUzUxMiJ9.eyJpZCI6IjY3YzgwZThjLWIxYTAtNDg2MC04MTNlLTJkMTJkMDAwMGMxNyIsImRhdGEiOnt9LCJyYW5kb20iOiIyMTAxOTJhOTdmOGZhM2JlNDc0Y2U3OTRkZDY4ZTE3ZCJ9.SauOXEheZWkIThKnsAwjMLR9Ro_InnekWZg-omxso8zNI0dfqORLcmSH2mJc3OhIViB0T393oOtqc6KFMMNUxA, accessed on 19 September 2025, and reference number [30]. The code for automated analysis is available in the GitHub (https://github.com/zgq2020/Liu-et-al.-2025, version 1.0, accessed on 1 August 2025).

## Supplementary Materials

The following supporting information can be downloaded at: https://www.mdpi.com/article/10.3390/insects16100997/s1, Figure S1: The photobehavior of female mosquitoes under different wavelengths of UV light; Figure S2: Video recordings and automated analysis were conducted to assess mosquito photobehavior under 345 nm UV light at an intensity of 150 μW/cm^2^; Figure S3: RNAi-mediated knockdown efficiency of *Ae. aegypti* opsin genes; Figure S4: RNAi-mediated knockdown efficiency of *Cx. quinquefasciatus* opsin genes; Table S1: Summary of experimental conditions for all behavioral assays; Table S2: qPCR primers of opsin genes used in expression profile; Table S3: Primers for dsRNA synthesis and detection; Table S4: Details of the test methods used for each sample analysis; Table S5: The results of multiple comparisons test for testing among different groups; Video S1: Representative recording of mosquito photobehavior; Video S2: Automated analysis of mosquito activity.

## Abbreviations

The following abbreviations are used in this manuscript:

*Ae. aegypti*	*Aedes aegypti*
*Ae. albopictus*	*Aedes albopictus*
*Cx. quinquefasciatus*	*Culex quinquefasciatus*
dsGFP	Double-strand green fluorescent protein
dsRNA	Double-strand RNA
min	Minute
PI	Preference index
RNAi	RNA interference
RT-qPCR	Quantitative real-time polymerase chain reaction
UV	Ultraviolet
ZT	Zeitgeber time
