# Supplementary material for: Elimination of Ultraviolet Light-Mediated Attraction Behavior in Culex Mosquitoes via dsRNA-Mediated Knockdown of Opsins
Source: Insects. 2025 Sep 25;16(10):997. doi: 10.3390/insects16100997 (PMC12563000; doi:10.3390/insects16100997)
Supplement: Supplementary file 1 [file insects-16-00997-s001.zip › insects-3850282-supplementary.pdf]

insects

MDPI

Supplementary Information

Elimination of ultraviolet light-mediated attraction behavior in *Culex* mosquitoes via dsRNA-mediated knockdown of Opsins

Table S1. Summary of experimental conditions for all behavioral assays.

Figure	Assay type	Mosquito species	Light wavelength (nm)	Light intensity (μW/cm²)	Biological replicates	Total number of mosquitoes tested	Test duration (min)
Fig. 1B	Y-maze assay	*Cx. quinquefasciatus*	345	50 μW/cm²	3	150	5
			345	150 μW/cm²	3	150	5
			345	500 μW/cm²	3	150	5
			345	1000 μW/cm²	3	150	5
			345	1500 μW/cm²	3	150	5
Fig. 1C	Y-maze assay	*Ae. albopictus*	345	50 μW/cm²	6	300	5
			345	150 μW/cm²	6	300	5
			345	500 μW/cm²	6	300	5
			345	1000 μW/cm²	4	200	5
			345	1500 μW/cm²	3	150	5
Fig. 1D	Y-maze assay	*Ae. aegypti*	345	5 μW/cm²	3	150	5
			345	10 μW/cm²	3	150	5
			345	50 μW/cm²	3	150	5
			345	50 μW/cm²	3	150	5
			345	500 μW/cm²	3	150	5
			345	1000 μW/cm²	3	150	5
Fig. 1E	Y-maze assay	Field *Cx. quinquefasciatus*	345	50 μW/cm²	3	150	5
			345	150 μW/cm²	3	150	5
			345	500 μW/cm²	3	150	5
			345	1000 μW/cm²	3	150	5
			345	1500 μW/cm²	3	150	5
Fig. 2B	Tube assay	*Ae. albopictus*	345	50 μW/cm²	3	218	5
		Cx. quinquefasciatus	345	50 μW/cm²	4	200	5
		Field *Cx. quinquefasciatus*	345	50 μW/cm²	3	150	5
		Ae. aegypti	345	50 μW/cm²	3	116	5
		Ae. albopictus	395	50 μW/cm²	3	150	5

Fig. 2C	Tube assay	*Cx. quinquefasciatus*	395	50 μW/cm²	3	150	5
		Field *Cx. quinquefasciatus*	395	50 μW/cm²	3	150	5
		Ae. aegypti	395	50 μW/cm²	3	111	5
Fig. 3C	Automated video-tracking	*Ae. albopictus*	345	50 μW/cm²	5	400	2
		Cx. quinquefasciatus	345	50 μW/cm²	6	480	2
		Field *Cx. quinquefasciatus*	345	50 μW/cm²	4	320	2
Fig. 3D	Automated video-tracking	*Ae. albopictus*	345	50 μW/cm²	5	400	2
		Cx. quinquefasciatus	345	50 μW/cm²	6	480	2
		Field *Cx. quinquefasciatus*	345	50 μW/cm²	4	320	2
Fig. 3E	Automated video-tracking	*Ae. albopictus*	345	50 μW/cm²	5	400	2
		Cx. quinquefasciatus	345	50 μW/cm²	6	480	2
		Field *Cx. quinquefasciatus*	345	50 μW/cm²	4	320	2
Fig. 3F	Automated video-tracking	*Ae. albopictus*	345	50 μW/cm²	5	400	2
		Cx. quinquefasciatus	345	50 μW/cm²	6	480	2
		Field *Cx. quinquefasciatus*	345	50 μW/cm²	4	320	2
Fig. 4B	Y-maze assay	*Ae. aegypti* dsGFP	345	10 μW/cm²	4	148	5
		ds*AaegOpsin1*	345	10 μW/cm²	3	109	5
		ds*AaegOpsin2*	345	10 μW/cm²	3	98	5
		ds*AaegOpsin8*	345	10 μW/cm²	4	144	5
		ds*AaegOpsin9*	345	10 μW/cm²	3	107	5
Fig. 5B	Y-maze assay	*Cx. quinquefasciatus* ds*GFP*	345	500 μW/cm²	5	232	5
		ds*CqOpsin2*	345	500 μW/cm²	5	180	5
		ds*CqOpsin3*	345	500 μW/cm²	5	176	5
		ds*CqOpsin5*	345	500 μW/cm²	5	198	5
		ds*CqOpsin6*	345	500 μW/cm²	5	189	5
		ds*CqOpsin11*	345	500 μW/cm²	5	183	5
Fig. 5C	Y-maze assay	*Cx. quinquefasciatus* ds*GFP*	345	50 μW/cm²	5	197	5
		ds*CqOpsin2*	345	50 μW/cm²	5	203	5
		ds*CqOpsin3*	345	50 μW/cm²	5	214	5
		ds*CqOpsin5*	345	50 μW/cm²	5	208	5
		ds*CqOpsin6*	345	50 μW/cm²	5	198	5
		ds*CqOpsin11*	345	50 μW/cm²	5	196	5
		ds*GFP*	345	500 μW/cm²	3	112	5
		ds*CqOpsin2*	345	500 μW/cm²	3	112	5

Fig. 5D	Y-maze assay	Field *Cx. quinquefasciatus*	ds*CqOpsin3*	345	500 µW/cm²	3	113	5
			ds*CqOpsin5*	345	500 µW/cm²	3	128	5
			ds*CqOpsin6*	345	500 µW/cm²	3	120	5
			ds*CqOpsin11*	345	500 µW/cm²	3	128	5
Fig. 5E	Tube assay	*Cx. quinquefasciatus*	ds*GFP*	345	500 µW/cm²	4	180	5
			ds*CqOpsin2*	345	500 µW/cm²	4	179	5
			ds*CqOpsin3*	345	500 µW/cm²	4	187	5
			ds*CqOpsin5*	345	500 µW/cm²	4	166	5
			ds*CqOpsin6*	345	500 µW/cm²	4	180	5
			ds*CqOpsin11*	345	500 µW/cm²	4	157	5
Fig. S1A	Y-maze assay	*Ae. albopictus*		345	150 µW/cm²	3	218	5
		Cx. quinquefasciatus		345	150 µW/cm²	3	150	5
		Field *Cx. quinquefasciatus*		345	150 µW/cm²	3	150	5
Fig. S1B	Tube assay	*Ae. albopictus*		345	50 µW/cm²	3	150	5
		Cx. quinquefasciatus		345	50 µW/cm²	3	150	5
		Field *Cx. quinquefasciatus*		345	50 µW/cm²	3	150	5
		Ae. aegypti		345	50 µW/cm²	3	150	5
Fig. S2A	Automated video-tracking	*Ae. albopictus*		345	150 µW/cm²	5	400	2
		Cx. quinquefasciatus		345	150 µW/cm²	8	640	2
		Field *Cx. quinquefasciatus*		345	150 µW/cm²	6	480	2
Fig. S2B	Automated video-tracking	*Ae. albopictus*		345	150 µW/cm²	5	400	2
		Cx. quinquefasciatus		345	150 µW/cm²	8	640	2
		Field *Cx. quinquefasciatus*		345	150 µW/cm²	6	480	2
Fig. S2C	Automated video-tracking	*Ae. albopictus*		345	150 µW/cm²	5	400	2
		Cx. quinquefasciatus		345	150 µW/cm²	8	640	2
		Field *Cx. quinquefasciatus*		345	150 µW/cm²	6	480	2
Fig. S2D	Automated video-tracking	*Ae. albopictus*		345	150 µW/cm²	5	400	2
		Cx. quinquefasciatus		345	150 µW/cm²	8	640	2
		Field *Cx. quinquefasciatus*		345	150 µW/cm²	6	480	2

Table S2. qPCR primers of opsin genes used in expression profile.

Primers for SYBR Green RT-qPCR	Forward Primer	Reverse Primer
CqOpsin1	CCTTATTCGGATGTTGCTCG	GGAGTT-GGTCAGCGGTTTAC
CqOpsin2	TGCCTTCGGAGATAAGACC	TGACTGATGGCGTAAACCC
CqOpsin3	GAACCACGAGAAGGCACTT	CACAAACAG-GAAACAGACGG
CqOpsin4	TGGAAGTGGAAGGGAAAG	AGCAGTTGACGCTGTTGA
CqOpsin5	ACAACTCTTCCAACGACTCC	TCCAGTGTGTGCTGTCTTG
CqOpsin6	GAACAGATGCGTGAACAGG	TGAACCACAGGGAAATGG
CqOpsin7	GTTATCGGCAACGGATGT	ATCAGGAAGTCGGA-GAAGG
CqOpsin8	AAGCCGATGACCAACAAC	CGAAGAATGGAGGGA-TAGTC
CqOpsin9	CGTCTCCATCTGGTCAATG	GTTGTTGGTCATCGGCTT
CqOpsin10	CCTCTGGTTTATGGCTTGG	ACTTGGGATGGCTGATTCC
CqOpsin11	TTTCGCTTATCGGGAACG	ATTGGCATCTCGCACATC
CqOpsin12	CTGCTCATCTGGTGTTACTCGT	TTCAGGGTTTGCGTTTCC
CqOpsin13	GCTTGCGAGGTCTACGCTAT	TCAGCCGAGATACCGTT-GAC
AaegOpsin1	CGGCTTTGTCTTCGAGTCTG	ACAAATGCCGCCATTC-TATTTC
AaegOpsin2	AGTAGTTCGTTCGCTGTGGG	GGCAGCCATTGTCTTCTCCT
AaegOpsin3	CCGATTACCCCACTGGCTAC	GAACTTCTGG-TACAGGGCGG
AaegOpsin4	GATCCTGCCGACGATACGC	AAGTGCCTTAAGCAGAT-TCCG
AaegOpsin5	CTGCCGACGATTCGCAGTC	TGTCCCTTTAAGCAGAT-TCCGTC
AaegOpsin7	ACAATCCGATCGTGTACGGG	AGCCATCGACTTAG-CATCGG
AaegOpsin8	CCTTCGTGAACAGGCCAAGA	GGCTATCCTCAC-CTCAACCG
AaegOpsin9	ACGATTCGGATACTCGCGTC	CATGCTT-GCTCACATGACCG
AaegOpsin10	TTCGCGCTATACGAGGAACG	GGATTGGTCTCGGTCAGGTC
AaegOpsin12	CGCTGATGTGCAAGGATGTG	TGTGAACGGGTTGCCGA-TAA

Table S3. Primers for dsRNA synthesis and detection.

**Primers for dsRNAsynthesis**	**Forward Primer**	**Reverse Primer**
dsRNA GFP	TAATACGACTCACTATAGGGCAACGGTGTGGACTTTGAC	TAATACGACTCACTATAGGGCGGCTGGTTCTTCAGATAGTT
dsRNA *CqOpsin2*	TAATACGACTCACTATAGGGTGTTTCTGCTCAACGAAACCG	TAATACGACTCACTATAGGGATCACAACGAACAGCGATGC
dsRNA *CqOpsin3*	TAATACGACTCACTATAGGGTGGTTTGGGCGTACACCATT	TAATACGACTCACTATAGGGAACCACGGTAGTCGCTTCTG
dsRNA *CqOpsin5*	TAATACGACTCACTATAGGGCGGTATGTCCCAGAGGGTAA	TAATACGACTCACTATAGGGTCGTTGGAAGAGTTGTCCG
dsRNA *CqOpsin6*	TAATACGACTCACTATAGGGACAGTGGTGCTATGGTCAAG	TAATACGACTCACTATAGGGGGTGTCGTTGGAAGAGTTG
dsRNA *CqOpsin11*	TAATACGACTCACTATAGGGCGGTGGAGCAATCAGCAATG	TAATACGACTCACTATAGGGGTCTAGGCAGGACACAACCT
dsRNA *AaegOpsin1*	TAATACGACTCACTATAGGGAGTTCCCACCGATGAATCC	TAATACGACTCACTATAGGGGGCGTAAACGATGATGTAGG
dsRNA *AaegOpsin2*	TAATACGACTCACTATAGGGTATGATTCACCCGCACTG	TAATACGACTCACTATAGGGGAAGATGGCGTAGATGATGA
dsRNA *AaegOpsin8*	TAATACGACTCACTATAGGGTTGTCAGTAGGCACCGTGA	TAATACGACTCACTATAGGGAGTGAACCGATGAAAGCG
dsRNA *AaegOpsin9*	TAATACGACTCACTATAGGGGTATCTTGCGACGGTGTTC	TAATACGACTCACTATAGGGGCGATGACTGCGTTAGTTATG
**Primers for SYBR Green RT-qPCR**	**Forward Primer**	**Reverse Primer**
*CqOpsin*2	CTGTTTGTCTGCGCTTGGAC	GCACGAAACAATCTTGCAGC
*CqOpsin*3	TATCGGCCGAAACTCGCTAC	AGGGCGAAAATTGTGAACGC
*CqOpsin*5	TACCGCTCTAGACGTTGTGA	ATGTCCACGACGGACATGTTG
*CqOpsin*6	ACATCTCCACGTGCAGTTTCTTA	GGTCAAGCGCGTGATTGTTG
*CqOpsin*11	GCCATCAGCCATCCGAAGTA	CTGCCGGAGGATCCGATATG

AaegOpsin1	CGGCTTTGTCTTCGAGTCTG	ACAAATGCCGCCATTCTATTTC
AaegOpsin2	AGTAGTTCGTTCGCTGTGGG	GGCAGCCATTGTCTTCTCCT
AaegOpsin8	CCTTCGTGAACAGGCCAAGA	GGCTATCCTCACCTCAACCG
AaegOpsin9	ACGATTCGGATACTCGCGTC	CATGCTTGCTCACATGACCG

Table S4. Details of the test methods used for each sample analysis

Figure	Test method	Sample	*t*	*df*	*P*
Fig. 1B	One sample *t* test	50 μW/cm²	10.36	5	< 0.001
		150 μW/cm²	12.14	5	< 0.001
		500 μW/cm²	17.23	5	< 0.001
		1000 μW/cm²	15.78	5	< 0.001
		1500 μW/cm²	1.323	5	0.2431
Fig. 1C	One sample *t* test	50 μW/cm²	10.65	11	< 0.001
		150 μW/cm²	7.326	11	< 0.001
		500 μW/cm²	9.629	11	< 0.001
		1000 μW/cm²	4.046	7	0.0049
		1500 μW/cm²	1.518	5	0.1896
Fig. 1D	One sample *t* test	5 μW/cm²	8.464	5	< 0.001
	One sample *t* test	10 μW/cm²	10.20	5	< 0.001
	Wilcoxon signed-rank test	50 μW/cm²	*T* = 21, *N* = 6		0.2810
	One sample *t* test	150 μW/cm²	0.8902	5	0.4141
	One sample *t* test	500 μW/cm²	0.9807	5	0.3718
	One sample *t* test	1000 μW/cm²	0.1514	5	0.8856
Fig. 1E	One sample *t* test	50 μW/cm²	18.27	5	< 0.001
		150 μW/cm²	14.70	5	< 0.001
		500 μW/cm²	11.67	5	< 0.001
		1000 μW/cm²	7.018	5	< 0.001
		1500 μW/cm²	0.5929	5	0.579
Fig. 2B	One sample *t* test	*Ae. albopictus*	9.985	5	< 0.001
		Cx. quinquefasciatus	14.32	7	< 0.001
		Field *Cx. quinquefasciatus*	12.25	5	< 0.001
		Ae. aegypti	1.895	5	0.1166
Fig. 2C	One sample *t* test	*Ae. albopictus*	14.23	5	< 0.001
	One sample *t* test	*Cx. quinquefasciatus*	5.098	5	0.0038
	One sample *t* test	Field *Cx. quinquefasciatus*	3.677	5	0.0143
	Wilcoxon signed-rank test	*Ae. aegypti*	*T* = 9, *N* = 6		0.4375
Fig. 3C	One sample *t* test	*Ae. albopictus*	8.625	5	< 0.001
		Cx. quinquefasciatus	7.207	5	< 0.001
		Field *Cx. quinquefasciatus*	6.608	5	< 0.001
Fig. 3D	Unpaired *t* test	UV vs Dark (*Ae. albopictus*)	7.377	18	< 0.001
		UV vs Dark (*Cx. quinquefasciatus*)	5.615	22	< 0.001

		UV vs Dark (Field *Cx. quinquefasciatus*)	9.247	14	< 0.001
Fig. 3E	Unpaired *t* test	UV vs Dark (*Ae. albopictus*)	6.691	18	< 0.001
		UV vs Dark (*Cx. quinquefasciatus*)	5.884	22	< 0.001
		UV vs Dark (Field *Cx. quinquefasciatus*)	9.416	14	< 0.001
Fig. 3F	Unpaired *t* test	UV vs Dark (*Ae. albopictus*)	4.053	18	< 0.001
		UV vs Dark (*Cx. quinquefasciatus*)	3.444	22	0.0023
		UV vs Dark (Field *Cx. quinquefasciatus*)	0.6237	14	0.5371
Fig. 4B	One sample *t* test	ds*AaegOpsin1*	11.25	5	< 0.001
	One sample *t* test	ds*AaegOpsin2*	13.34	5	< 0.001
	One sample *t* test	ds*AaegOpsin8*	7.423	5	< 0.001
	One sample *t* test	ds*AaegOpsin9*	6.633	5	< 0.001
	Wilcoxon signed-rank test	ds*GFP*	*T* = 36, *N* = 8		0.0078
Fig. 5B	One sample *t* test	ds*GFP*	10.89	9	< 0.001
		CqOpsin2	11.52	9	< 0.001
		CqOpsin3	3.377	9	0.0082
		CqOpsin5	0.9304	9	0.3764
		CqOpsin6	1.426	9	0.1876
		CqOpsin11	9.955	9	< 0.001
Fig. 5C	One sample *t* test	ds*GFP*	10.32	9	< 0.001
	Wilcoxon signed-rank test	*CqOpsin2*	*T* = 53, *N* = 10		0.0039
	One sample *t* test	*CqOpsin3*	0.07227	9	0.944
	One sample *t* test	*CqOpsin5*	0.4328	9	0.6753
	One sample *t* test	*CqOpsin6*	1.392	9	0.1975
	One sample *t* test	*CqOpsin11*	7.479	9	< 0.001
Fig. 5D	One sample *t* test	ds*GFP*	5.003	5	0.0041
		CqOpsin2	5.464	5	0.0028
		CqOpsin3	1.101	5	0.3212
		CqOpsin5	0.5257	5	0.6216
		CqOpsin6	0.4230	5	0.6899
		CqOpsin11	14.27	5	< 0.001
Fig. 5E	One sample *t* test	ds*GFP*	8.874	7	< 0.001
		CqOpsin2	6.691	7	< 0.001
		CqOpsin3	1.596	7	0.1546
		CqOpsin5	1.275	7	0.2429

		CqOpsin6	3.202	7	0.015
		CqOpsin11	11.64	7	< 0.001
Fig. S1A	One sample *t* test	*Ae. albopictus*	5.944	5	< 0.001
		Cx. quinquefasciatus	8.450	5	< 0.001
		Field *Cx. quinquefasciatus*	21.27	5	< 0.001
Fig. S1B	One sample *t* test	*Ae. albopictus*	8.638	5	< 0.001
		Cx. quinquefasciatus	4.628	5	0.0057
		Field *Cx. quinquefasciatus*	9.004	5	< 0.001
		Ae. aegypti	40.73	5	< 0.001
Fig. S2A	One sample *t* test	*Ae. albopictus*	11.42	9	< 0.001
		Cx. quinquefasciatus	5.206	15	< 0.001
		Field *Cx. quinquefasciatus*	8.288	11	< 0.001
Fig. S2B	Unpaired *t* test	UV vs Dark (*Ae. albopictus*)	11.54	18	< 0.001
		UV vs Dark (*Cx. quinquefasciatus*)	6.473	30	< 0.001
		UV vs Dark (Field *Cx. quinquefasciatus*)	5.844	22	< 0.001
Fig. S2C	Unpaired *t* test	UV vs Dark (*Ae. albopictus*)	9.816	18	< 0.001
		UV vs Dark (*Cx. quinquefasciatus*)	6.227	30	< 0.001
		UV vs Dark (Field *Cx. quinquefasciatus*)	5.402	22	< 0.001
Fig. S2D	Mann Whitney *U* test	UV vs Dark (*Ae. albopictus*)	$U = 10, N_1 = N_2 = 10$		0.0015
	Unpaired *t* test	UV vs Dark (*Cx. quinquefasciatus*)	4.227	30	
	Mann Whitney *U* test	UV vs Dark (Field *Cx. quinquefasciatus*)	$U = 10, N_1 = N_2 = 12$		0.5137
Fig. S3A	Unpaired *t* test	*dsGFP* vs. *dsOpsin1*	11.31	18	< 0.001
Fig. S3B	Unpaired *t* test	*dsGFP* vs. *dsOpsin2*	9.599	18	< 0.001
Fig. S3C	Unpaired *t* test	*dsGFP* vs. *dsOpsin8*	5.852	18	< 0.001
Fig. S3D	Unpaired *t* test	*dsGFP* vs. *dsOpsin9*	7.152	18	< 0.001
Fig. S4A	Mann-Whitney *U* test	*dsGFP* vs. *dsOpsin2*	$U = 9, N_1 = N_2 = 10$		0.0011
Fig. S4B	Mann-Whitney *U* test	*dsGFP* vs. *dsOpsin3*	$U = 3, N_1 = N_2 = 10$		< 0.001
Fig. S4C	Mann-Whitney *U* test	*dsGFP* vs. *dsOpsin5*	$U = 10, N_1 = N_2 = 10$		0.0015
Fig. S4D	Unpaired *t* test	*dsGFP* vs. *dsOpsin6*	2.928	18	0.009
Fig. S4E	Unpaired *t* test	*dsGFP* vs. *dsOpsin11*	4.278	18	< 0.001

"*t*" represents the test statistic value of the one sample *t* test, "*df*" respresents the degrees of freedom, "*T*" represents the test statistic of the Wilcoxon signed-ranks test, "*N*" respresents the sample size, and "*U*" represents the test statistic value of the Mann-Whitney *U* test.

Table S5. The results of multiple comparisons test for testing among different groups.

Figure	Test methods	Groups	Mean rank diff.	*P*
Fig. 4B	Kruskal-Wallis test		H_4= 7.53	0.1104
	Dunn's multiple comparisons test	*dsGFP* vs. *dsOpsin1*	-3.458	> 0.9999
		dsGFP vs. *dsOpsin2*	-4.042	> 0.9999
		dsGFP vs. *dsOpsin8*	8.500	0.8712
		dsGFP vs. *dsOpsin9*	2.542	> 0.9999
Fig. 5B	One-way ANOVA		*F* (5, 54) = 20.39	< 0.001
	Tukey's multiple comparisons test	*dsGFP* vs. *dsOpsin2*	-0.08493	0.7313
		dsGFP vs. *dsOpsin3*	-0.3485	< 0.001
		dsGFP vs. *dsOpsin5*	-0.4367	< 0.001
		dsGFP vs. *dsOpsin6*	-0.4408	< 0.001
		dsGFP vs. *dsOpsin11*	-0.1088	0.4841
Fig. 5C	Kruskal-Wallis test		H_5 = 33.44	< 0.001
	Dunn's multiple comparisons test	*dsGFP* vs. *dsOpsin2*	-5.15	> 0.9999
		dsGFP vs. *dsOpsin3*	-31.15	0.001
		dsGFP vs. *dsOpsin5*	-29	0.0031
		dsGFP vs. *dsOpsin6*	-26.45	0.0106
		dsGFP vs. *dsOpsin11*	-4.25	> 0.9999
Fig. 5D	One-way ANOVA		*F* (5, 30) = 6.693	< 0.001
	Tukey's multiple comparisons test	*dsGFP* vs. *dsOpsin2*	-0.04459	0.9976
		dsGFP vs. *dsOpsin3*	-0.3146	0.0412
		dsGFP vs. *dsOpsin5*	-0.3538	0.0162
		dsGFP vs. *dsOpsin6*	-0.3564	0.0152
		dsGFP vs. *dsOpsin11*	0.01875	> 0.9999
Fig. 5E	One-way ANOVA		*F* (5, 42) = 13.04	< 0.001
	Tukey's multiple comparisons test	*dsGFP* vs. *dsOpsin2*	-0.0104	> 0.9999
		dsGFP vs. *dsOpsin3*	-0.308	< 0.001
		dsGFP vs. *dsOpsin5*	-0.3209	< 0.001
		dsGFP vs. *dsOpsin6*	-0.2455	0.0054
		dsGFP vs. *dsOpsin11*	0.02093	0.9995

"*H*" represents the test statistic value of the Kruskal-Wallis test and "*F*" represents the test statistic value of the One-way ANOVA.

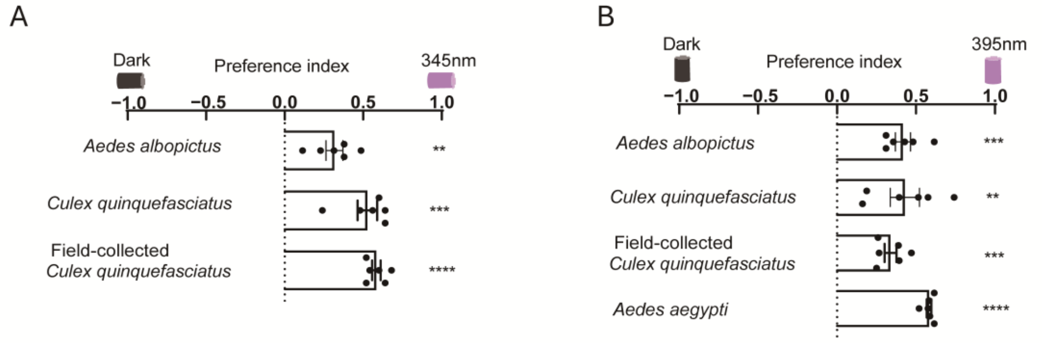

Figure. S1 The photobehaviour of female mosquitoes under different wavelengths of UV light. (A) photobehaviour of laboratory-reared *Ae. albopictus*, *Cx. quinquefasciatus* and field-collected *Cx. quinquefasciatus* under 345 nm UV light at intensities of 150 μW/cm². (B) photobehaviour of laboratory-reared *Ae. albopictus*, *Cx. quinquefasciatus*, *Ae. aegypti*, and field-collected *Cx. quinquefasciatus* under 395 nm UV light at intensities of 50 μW/cm². (A-B) Values represent mean ± SEM of ≥3 biological replicates (two technical replicates each). The total number of female mosquitoes tested in each group is presented in Table S1. Statistical significance of phototaxis relative to chance was computed using a one-sample *t*-test. Significance: ***P* < 0.01, ****P* < 0.001, *****P* < 0.0001.

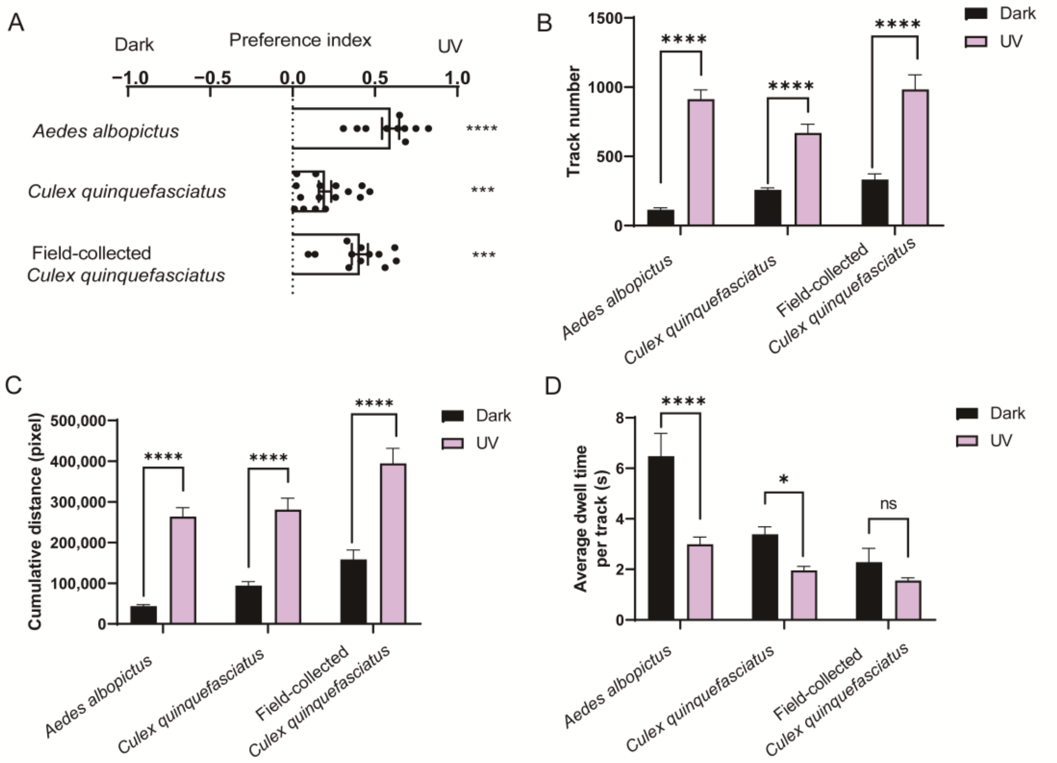

Figure S2. Video recordings and automated analysis were conducted to assess mosquito photobehaviour under 345 nm UV light at an intensity of 150μW/cm². (A-D) Photopreference between darkness and 345nm UV light of 150 μW/cm². (A) The preference index was calculated by summing the PI values for each frame and dividing by the total number of frames. (B) The number of mosquito tracks presented at respective window. (C) The cumulative distance mosquitoes moved on respective mesh window of all tracks. (D) The average dwell time that each mosquito track spent on respective mesh window. (A-D) Values represent mean ± SEM of ≥3 biological replicates (two technical replicates each). The total number of female mosquitoes tested in each group is presented in Table S1. (A) Data were analyzed using one sample *t*-test. (B-D) Group comparisons were performed using unpaired *t*-test. or Mann-Whitney test. Significance: **P* < 0.05, ****P* < 0.001, *****P* < 0.0001, ns: not significant.

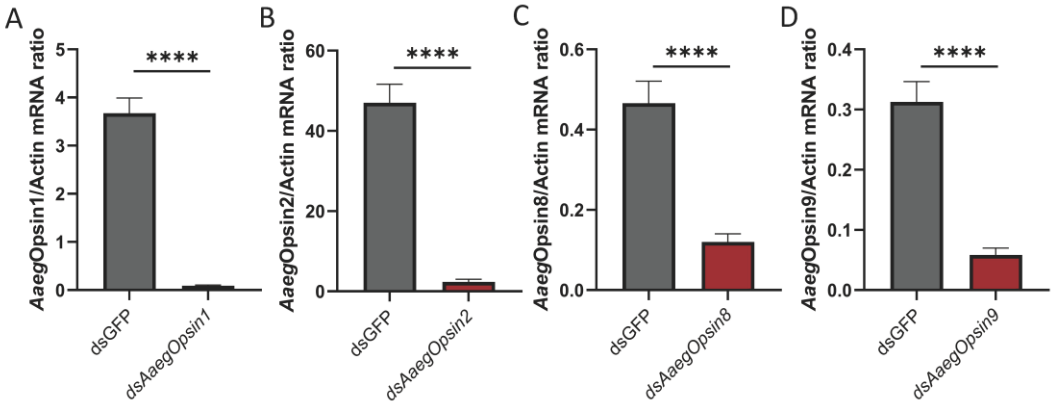

Figure S3. RNAi-mediated knockdown efficiency of *Ae. aegypti* opsin genes. Gene expression levels were quantified by RT-qPCR following intrathoracic injection of species-specific dsRNA. Expression of (A) *AaegOpsin1* after ds*AaegOpsin1* treatment, (B) *AaegOpsin2* after ds*AaegOpsin2* treatment, (C) *AaegOpsin8* after ds*AaegOpsin8* treatment, and (D) *AaegOpsin9* after ds*AaegOpsin9* treatment. Each group contained 30 female mosquitoes. (A-D) Values represent mean ± SEM of ≥3 biological replicates. Expression levels of opsin genes were normalized against *Ae. aegypti* Actin (*AAEL011197*). Group comparisons were performed using unpaired *t*-test. Significance: ****$P <$ 0.0001.

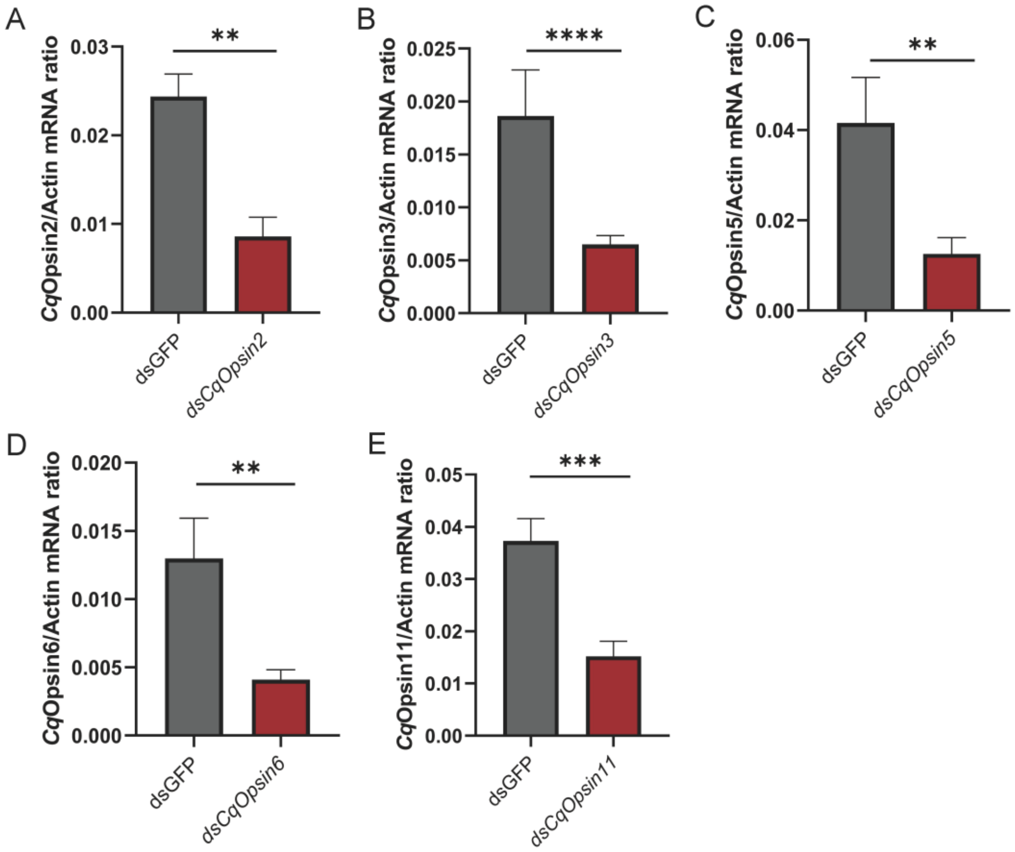

Figure S4. RNAi-mediated knockdown efficiency of *Cx. quinquefasciatus* opsin genes. Gene expression levels were quantified by RT-qPCR following intrathoracic injection of species-specific dsRNA. Expression of (A) *CqOpsin2* after ds*CqOpsin2* treatment, (B) *CqOpsin3* after ds*CqOpsin3* treatment, (C) *CqOpsin5* after ds*CqOpsin5* treatment, (D) *CqOpsin6* after ds*CqOpsin6* treatment, and (E) *CqOpsin11* after ds*CqOpsin11* treatment. Each group contained 30 female mosquitoes. (A-E) Values represent mean ± SEM of ≥3 biological replicates. Expression levels of opsin genes were

normalized against *Cx. quinquefasciatus* Rpl8 (*LOC6031076*) (A-C) Statistical comparisons between groups were conducted using Mann-Whitney test. (D-E) Statistical comparisons between groups were conducted using unpaired t-test. Significance: $^{**}P < 0.01$, $^{***}P < 0.001$, $^{****}P < 0.0001$.

Video S1. Representative recording of mosquito photobehavior. This video documents the behavioral responses of mosquitoes exposed to 345 nm UV illumination, delivered from a computer-controlled tunable light source directed toward the right mesh windows. The footage spans 2 minutes and is presented at real-time speed without modification or acceleration.

Video S2. Automated analysis of mosquito activity. The video shows an automated program that records and analyzes mosquito activity. We used an automated object-tracking and PI calculating script based on MATLAB, to analyze mosquitoes in recorded video.
